# Kentucky Institution for the Education of the Blind

**Published:** 1845-03

**Authors:** 


KENTUCKY INSTITUTION FOR THE EDUCATION OF THE BLIND.

The '*Third Annual Report*' of this interesting establishment has just been laid on our table. It presents much to interest every benevolent mind. Within the past year, the Board of Visitors have erected a capacious, commodious, and beautiful edifice, in the edge of Louisville, which will be finished in the course of the ensuing Spring.

The number of pupils is 22, varying in age from 9 to 24 years. They are contented and make good progress in spelling, reading, writing, arithmetic, music, and various handicraft arts. They, in general, enjoy good health. Indeed, it has been found that the health of almost every pupil, has improved since coming into the institution—the beneficial result of regular training, in regard to study, exercise, diet, and sleep. Why is it, that so few of the numerous blind persons of the State, are found in this most eligible asylum? We fear, that our brethren do not very faithfully discharge their duty, towards those whom they have been unable to protect or restore from blindness. The people need instruction and exhortation; and who are so well qualified to impart both, as their family physicians? To aid them in this matter, we mention from the Report, that the annual expense of a pupil is one hundred dollars, payably quarterly in advance. This covers board, lodging, washing, tuition, music, books, and stationary; and certainly it is low. No child can be admitted under six nor over fifteen years of age: except by special permission of the Board. Indigent children, of residents of the State of Kentucky, are admitted, supported and taught *gratuitously;* provided it be avouched by a magistrate or some other respectable person, that neither the parents nor the near relations, are able to meet the expense.

In every instance of application for admission, answers must be made to the following questions: 1. *What is the name of the applicant?* 2. *When and where born?* 3. *What are the names of the parents?* 4. *Are they living?* 5. *What is the name of the post-office nearest to their residence?* 6. *What are the pecuniary circumstances of the parents and relations?* 7. *Is the blindness total?* 8. *If not, what degree of vision remains?* 9. *How was the blindness produced?* 10. *Is the applicant of good natural capacity, and free from bodily defects, and offensive and infectious diseases?* 11. *Are there other instances of blindness in the same family, or among their relations?*

All communications must be directed, post-paid, to Mr. Bryce M. Patten, Director. Louisville, Ky.

Anxious to give our notice a practical character, we have transcribed the terms and conditions of admission, that our brethren, throughout the State, may have and impart the information, necessary to correct action, by those who may desire to place their children in the institution.

We may add, for the benefit of those who live out of Kentucky, that pupils from other States are admitted. There is already one from New Orleans. We hope there will be many more from different parts of the West and South; for the number who are now groping and vegetating in total darkness, is very great.

We are happy to learn, that a bill for the benefit of the institution, has passed the legislature of Kentucky. D.

